# Effect of the Specific Carbohydrate Diet on the Microbiome of a Primary Sclerosing Cholangitis and Ulcerative Colitis Patient

**DOI:** 10.7759/cureus.2177

**Published:** 2018-02-09

**Authors:** Alanna Dubrovsky, Christopher L Kitts

**Affiliations:** 1 School of Medicine, UC Davis School of Medicine; 2 Biological Sciences, California Polytechnic State University, San Luis Obispo

**Keywords:** fecal bacteria, ulcerative colitis, primary sclerosing cholangitis, specific carbohydrate diet, microbiome, 16s rrna gene sequencing

## Abstract

A 20-year-old female was diagnosed with ulcerative colitis (UC) at age 14 and primary sclerosing cholangitis (PSC) at age 16. The PSC was successfully treated with high doses of oral vancomycin; however, the UC was more difficult to manage. After many drug treatments failed to treat the UC, the patient began following the specific carbohydrate diet (SCD). This report documents fecal microbiome changes resulting from following the SCD for two weeks. The DNA extracted from fecal samples was subjected to 16S rRNA gene sequencing to quantify bacterial species abundance. Not only were substantial changes in the fecal bacterial composition detectable within two weeks, but all UC symptoms were also controlled as early as one week following the start of the diet. The patient's fecal microbiota was dramatically different from those of three healthy control subjects and showed remarkable loss of bacterial diversity in terms of species richness, evenness, and overall diversity measures. Other specific changes in bacterial composition included an increase in Enterobacteriaceae, including Escherichia and Enterobacter species. A two- to three-fold decrease was observed in the prevalence of the most dominant fecal bacterial species, Fusobacterium ulcerans, after two weeks on the SCD. Overall species diversity and evenness increased to levels near the controls, although species richness remained low. These findings provide information on the fecal bacteria from a patient with PSC and UC, following prolonged oral vancomycin treatment, and identifies a potentially specific microbial effect for the SCD.

## Introduction

Ulcerative colitis (UC) is a subtype of inflammatory bowel disease (IBD), characterized by immune dysregulation leading to chronic colonic inflammation. A combination of genetic factors and environmental triggers could result in the UC disease phenotype; however, both have proven difficult to identify. One of the environmental triggers could involve the intestinal microbiome composition [1].

Approximately 80-90% of primary sclerosing cholangitis (PSC) patients also suffer from UC [2]. The link between the two illnesses is largely unknown; however, a recent publication reveals it may involve T-cells of common clonal origin infiltrating the intestines and liver [3].

Over the past decade, the intestinal microbiome has become a subject of intense study. Naturally, IBD is one of the main diseases being analyzed for pathogenic intestinal microbiome involvement. Dietary supplements, probiotics, and various diets have all been investigated as possible therapies. The specific carbohydrate diet (SCD), originally described as a treatment for Celiac disease in the 1920s and applied to IBD in 1987, has recently made headlines with its potential ability to treat IBD [4]. The SCD is a very restrictive diet, limiting most disaccharides and starches, with the goal of decreasing bacterial species that both feed on malabsorbed carbohydrates and create acids and inflammatory byproducts. Most unprocessed fruits, vegetables, and meat products are acceptable, while all grains, starch-heavy foods such as potatoes, and soft dairy products such as store-bought yogurt and milk are eliminated.

A recent study (available as "Published Ahead-of-Print") by Suskind et al. [4] investigated the effects of the SCD on the microbiome of pediatric Crohn’s and UC patients. The study identified intestinal microbial changes in response to the SCD. The case presented here identifies additional potential microbial effects of the SCD in an adult UC and PSC patient.

## Case presentation

The patient discussed in this case report presented with symptoms of bloody diarrhea, frequent bowel movements, lower left quadrant abdominal pain, and fatigue in October 2009 at age 14. She received the diagnosis of ulcerative colitis (UC) one week following onset of symptoms subsequent to a colonoscopy and gastrointestinal mucosal biopsies. Numerous medications were prescribed in attempts to control symptoms, including mesalamine, VSL#3, Apriso, and hydrocortisone steroid enemas. Although each medication temporarily relieved symptoms, none maintained long-lasting effects. Treatment with Remicade was effective; however, injection reactions led the physician and patient to seek other treatment options. Subsequent treatment with Humira was ineffective.

Routine blood tests over a four-month time period, when the patient was 16, detected increasingly elevated gamma-glutamyl transpeptidase, alanine transaminase, and aspartate transaminase activity. primary sclerosing cholangitis (PSC) was suspected and confirmed via liver biopsy in March 2011. Vancomycin (500 mg, three times daily) was prescribed, and liver enzymes normalized. UC symptoms remained, however, leading to increased vancomycin dosages to 750 mg three times daily and finally 1000 mg vancomycin three times daily. It was concluded that while vancomycin treated PSC in this patient, it had no beneficial effect on UC, and Remicade was once again administered. The patient developed an allergic reaction to Remicade in 2012 and discontinued treatment, instead opting to treat UC using the specific carbohydrate diet (SCD). For one year the patient was treated using SCD, after which Remicade was once again prescribed at five mg/kg every eight weeks, with no allergic reaction. To investigate the mode of action of the SCD, Remicade was stopped for a two-week period in March 2015, during which the patient, now age 20, was once again treated with the SCD. 

Baseline stool samples (pre-diet 1, pre-diet 2) were collected two weeks prior to the initiation of the SCD, while the patient was still being treated with Remicaid, to serve as confirmation of microbiome composition before the SCD. A prescheduled Remicade treatment was delayed and the SCD was followed for two weeks, after which another stool sample was collected (post-diet). The two-week time period was sufficient for detectable microbiome changes in previous literature studies [4]. Samples from three healthy subjects (volunteer college-age students; one male, two female) were included as controls for comparison. Control subjects' antibiotic use history was not collected and no dietary data were collected. All samples were analyzed by high-throughput sequencing of the V4 region of the 16S rRNA gene, using an Ion Torrent Personal Genome Machine™ sequencer (MR-DNA labs, Shallowater, TX). Between 48,000 and 129,000 16S rRNA gene sequences passing quality control (QC) parameters were returned from the samples (Table 1) and assigned a taxonomic origin using the MR-DNA Lab’s 16S sequence analysis protocol.

The patient’s fecal microbiome was consistently less diverse than microbiomes from healthy subjects, with fewer total phyla, families, and species represented in the sequence collections, alongside much lower scores in a variety of diversity measures (Table 1). Intriguingly, the SCD treatment appeared to decrease the number of taxa represented in the post-diet sample. However, the post-diet bacterial community was more evenly distributed across multiple species as shown by Simpson’s and Shannon’s diversity indexes, as well as Pielou’s index of evenness (Table 1). Thus, overall diversity most likely increased post-diet and the decrease in total taxa is due to the low number of 16S sequences (less than half) from the post-diet sample as compared to other samples.

**Table 1:** Diversity data from microbiome sequence analysis of control and patient samples. Included information: total number of QC-passed 16S rRNA gene sequences, representation of bacterial taxa at the phylum, family and species level, and four measures of species diversity for each sample.

Analysis	Control 1	Control 2	Control 3	Pre-Diet 1	Pre-Diet 2	Post-Diet
16S Sequences	121,096	95,039	75,724	129,299	114,233	48,771
Phylum	11	6	9	8	8	4
Family	41	31	35	26	31	12
Species	132	123	119	65	68	55
d (Richness)	11.2	10.6	10.5	5.4	5.8	5.0
J' (Evenness)	0.68	0.69	0.72	0.25	0.45	0.72
Simpson's	0.94	0.95	0.95	0.37	0.70	0.91
Shannon's	3.31	3.33	3.44	1.05	1.88	2.87

Prior to SCD treatment, Fusobacterium ulcerans, family Fusobacteriaceae, dominated the patient’s microbiome, comprising 83.22% to 53.61% of the sequences returned per sample at the family level, and 51.67% to 79.11% at the species level. The next most common species in the pre-diet samples was Viellonella dispar, family Veillonellaceae, comprising 18.91% to 2.6% at the family level, and 14.30% to 1.37% at the species level. No species from either of these families were detected in any of the healthy subject’s samples, with the exception of Veillonellaceae in Control 1. In contrast, the control microbiomes were dominated by species from the Bacteroideaceae, Ruminococcaceae, and Lachnospiraceae families. The patient’s post-diet microbiome composition changed dramatically, with a decrease of Fusobacterium ulcerans to 23.11% and a marked increase in abundance of several species in the Enterobacteriaceae family (Tables 2-3).

**Table 2:** Relative abundance of 16S rRNA gene sequences (percent of total). Comparison of key family-level groupings between control and patient samples to highlight family-level differences in microbiome composition. Not all families detected were included in this table.

Family	Control 1	Control 2	Control 3	Pre-Diet 1	Pre-Diet 2	Post-Diet
Bacteroidaceae	23.98	40.34	17.02	0.00	0.00	0.06
Enterobacteriaceae	0.01	0.00	0.00	9.89	18.11	70.44
Erysipelotrichaceae	1.15	0.23	1.66	0.39	2.11	0.75
Fusobacteriaceae	0.00	0.00	0.00	83.22	53.61	25.47
Veillonellaceae	0.15	0.00	0.00	2.60	18.91	1.32

**Table 3:** Relative abundance of 16S rRNA gene sequences (percent of total). Comparison of key species among control and patient samples to highlight species-level differences in microbiome composition. Not all species detected were included in this table.

Genus species	Control 1	Control 2	Control 3	Pre-Diet 1	Pre-Diet 2	Post-Diet
Bacteroides spp.	1.30	3.49	0.81	0.00	0.00	0.06
Enterobacter hormaechei	0.00	0.00	0.00	2.89	5.11	10.17
Klebsiella oxytoca	0.00	0.00	0.00	0.00	0.00	3.69
Klebsiella pneumoniae	0.00	0.00	0.00	0.22	0.43	0.09
Salmonella enterica	0.00	0.00	0.00	0.18	0.34	0.64
Escherichia coli	0.00	0.00	0.00	0.42	1.47	5.38
Shigella flexneri	0.00	0.00	0.00	0.02	0.07	5.86
Coprobacillus cateniformis	0.00	0.00	0.00	0.00	0.03	0.75
Fusobacterium ulcerans	0.00	0.00	0.00	79.15	51.67	23.11
Veillonella dispar	0.14	0.00	0.00	1.37	14.30	1.05

## Discussion

While this patient’s IBD was complicated by PSC and vancomycin treatment, Lee et al. showed that Fusobacterium spp. can be isolated from IBD patients and specifically from colonic ulcers [5]. The data collected in this case study supports previous evidence that Fusobacterium may play a stimulatory role in IBD [6]. The therapeutic potential of the SCD is recorded in two observational studies assessing the effectiveness of the SCD [7], as well as an interventional study [4] analyzing patients’ microbiomes and serum biomarkers. Suskind et al. [4] observed immense variation in microbiomes across 12 pediatric IBD patients, as well as between patients’ pre- and post-dietary interventions. None of the patients in that study showed the changes in Fusobacterium spp. shown here. It is important to note that the diet was effective in decreasing symptoms in multiple patients, despite this variation [4]. Along with the Suskind et al. study, this case study supports the hypothesis that the SCD’s therapeutic actions stem from a change in microbiome composition. This case study identifies Fusobacterium ulcerans, a species not already noted in the literature as associated with IBD, as another potential target for dietary intervention. This study also documents the therapeutic success and intestinal microbial changes in an adult IBD patient as opposed to the pediatric patients in the Suskind et al. study.

Although Fusobacterium dramatically decreased in relative abundance following the SCD, they remained the dominant species in this patient’s microbiome. Thus, the subject's improved symptoms could be due in part to an increase in other microbial species, rather than a decrease in Fusobacterium. In addition, Veillonella remained among the dominant species in the post-diet sample, which is concerning since Kasai et al. report an increased risk of adenocarcinoma in the presence of both species [8]. In fact, Veillonella abundance was not clearly affected by the SCD, given the large pre-diet variation in Veillonella. This may be an important species to study in the future, considering its presence in many PSC patient microbiomes and rarity in healthy microbiomes. Despite a substantial post-diet increase in some species abundance, overall diversity did not rise to the level seen for control samples. While the short duration of the study and the patient’s antibiotic regimen may have limited visible effects, the SCD may not be able to fully correct IBD-related dysbiosis.

Importantly, the SCD is not an effective symptom management regimen for every IBD patient. Some patients do not experience symptom relief no matter the duration of treatment. This lack of response may be explained by their microbiome; if the species identified by Suskind et al. and this study are not present in a patient’s microbiome, they may not experience a therapeutic microbiome shift. However, as shown in this case and in five out of 12 patients in Suskind et al., dietary intervention can have substantial effects on clinical symptoms, serum inflammatory biomarkers, and microbiome composition after just two weeks.

The current leading drug treatments for IBD, including biologics and steroids, work downstream to decrease inflammation and thus treat symptoms. While Fusobacterium is not always present in IBD patients, it is present and increased in a large enough population that further investigation into the effects of the SCD on Fusobacterium is warranted and may contribute to upstream therapeutic agent development [9-10].

## Conclusions

The SCD was an effective therapy for IBD in this patient with IBD and PSC. Determining factors for SCD efficacy may involve the dominant intestinal bacterial species and may thus be patient specific, arguing for microbiome analysis as a diagnostic tool. While there are effective and potent downstream medications, it would be more advantageous to treat upstream causes of IBD. More research is needed to illuminate interactions between vancomycin dosing, PSC, microbial composition, and IBD symptoms and how they may differ between individual patients.
